# Incorporation of Fluorescent Fluorinated Methacrylate Nano-Sized Particles into Chitosan Matrix Formed as a Membranes or Beads

**DOI:** 10.3390/polym14132750

**Published:** 2022-07-05

**Authors:** Anna Szwajca, Sandra Juszczyńska, Maciej Jarzębski, Elżbieta Baryła-Pankiewicz

**Affiliations:** 1 Department of Chemistry, Adam Mickiewicz University in Poznań, Uniwersytetu Poznańskiego 8, 61-614 Poznań, Poland; sandra.juszczynska97@gmail.com; 2 Department of Physics and Biophysics, Faculty of Food Science and Nutrition, Poznan University of Life Sciences, Wojska Polskiego 38/42, 60-637 Poznań, Poland; maciej.jarzebski@up.poznan.pl; 3 Faculty of Health Sciences, Pomeranian Medical University in Szczecin, Żołnierska 48, 71-210 Szczecin, Poland; elzpan@gmail.com

**Keywords:** chitosan hydrogel, polymer nanocomposites, functional membrane, chitosan beads, Rhodamine B, fluorescent nanoparticles

## Abstract

Fluorescent particles are of particular interest as probes and active agents for biomedical, pharmaceutical, and food applications. Here, we present two strategies for incorporation of core-shell acrylic fluorescent nanoparticles (NPs) with Rhodamine B (RhB) as a dye into a chitosan (CS) matrix. We selected two variants of NPsRhB immobilisation in a CS membrane and biopolymeric CS beads. Modification of the method for production of the biopolymer cover/transporter of nanoparticles allowed two series of hydrogels loaded with nanoparticles to be obtained with a similar concentration of the aqueous solution of the nanoparticles. Microscopic analysis showed that the NPs were nonuniformly distributed in millimetre-sized CS beads, as well as membranes, but the fluorescence signal was strong. The composition of CS layers loaded with nanoparticles (CS/NPsRhB) showed water vapour barrier properties, characterised by the contact angle of 71.8°. Finally, we incorporated NPsRhBCS beads into a gelatine matrix to check their stability. The results confirmed good stability of the NPsRhBCS complex system, and no dye leakage was observed from the beads and the membranes. The proposed complex system demonstrated promising potential for further use in bioimaging and, thus, for the development of advanced diagnostic tools.

## 1. Introduction

Polymeric membranes are defined as thin semipermeable barriers between two media that permit permeation of certain types of particles, ions, or nanoparticles of specific properties (size, solubility, and diffusion rate). Membranes may be in the shape of a flat foil, tube, capillary or hollow fibre with porous walls, etc., depending on the specific application [1]. As much effort is directed towards making environmentally friendly and biodegradable products, the membranes made of a combination of synthetic, especially as a nanocomposite [2,3], and biodegradable polymers have become a dedicated subject of research [4,5,6,7]. Chitosan is a polymer of semi-synthetic origin characterised by multidimensional properties, highly refined functionality, and a wide range of applications [7]. To improve the effectiveness of chitosan, different fillers, such as carbon nanotubes (CNTs), functionalised CNT composites, fullerene, modified C60, or other nanocomposites, can be used. Thanks to the electrical, optical, and mechanical properties of these composites, they can enhance the performance of chitosan. A combination of polymers of different properties can lead to an inconsistent system or a system with new and improved properties. New properties can be checked using measurement techniques, such as infrared (IR) and ultraviolet–visible (UV-Vis) spectroscopies, contact angle (CA) measurement, and stereoscopic and fluorescent microscopies. New features of CS membranes are revealed in the processes of filtration and controlled transport of substances [8]. The chemical modification of natural polymer is very popular for obtaining new materials for analytical and medical applications. An example is the production and use of a per-fluorocarbon-conjugated chitosan hydrogel to provide oxygen [9]. Moreover, synthetic polymers are characterised by good mechanical strength and stability [9]. Combined with polymers, such as polysaccharides, polyesters, and proteins, they were considered promising membrane materials because of their biodegradability in the first place. Chitosan (CS), which is a copolymer, deserves special attention from among the derivatives of biodegradable polysaccharides for the production of membranes. It consists of ß-(1→4)-2-acetamido-2-deoxy-D-glucopyranose and ß-(1→4)-2-amino-2-deoxy-D-glucopyranose monomers, and the share of the latter compound in the structure of the polymer is ≥50% [10]. Chitosan is the product of chitin deacetylation reaction [11]. The process of deacetylation may take several hours in a 50% sodium hydroxide solution and at a high temperature or can proceed as a biological process of chitin treatment with a hydrolytic enzyme N-deacetylase [12]. Chitin is a natural polymer that occurs in marine invertebrates, such as crabs and molluscs, and is a major component of the insect exoskeleton. It is also a component of cell walls in some types of fungi [11]. Chitin is a ß-(1→4)-2-acetamido-2-deoxy-D-glucopyranose (in position 2,3-trans) homopolymer. Chitin chains form ordered structures that come in three polymorphic forms—α, ß, and γ. The α form of chitin is the most common. Chain units in the α form of chitin are arranged antiparallel with strong hydrogen bonds between the chains, which stabilise the polymorphic structure [11]. The poor chemical reactivity of chitin affects its application. Chitosan with a high degree of deacetylation (DD) (over 65%) shows a high density of positive charge in an acidic environment (below pH = 6.2), which makes it polycationic in nature [13]. Additionally, the presence of amine and hydroxyl groups affects the sorption properties of the copolymer [14]. CS macromolecules of different deacetylation degrees differ in the average molecular weight, breaking strength, hydrophilic properties, and reactivity. As a copolymer, it can be obtained in various forms, such as membranes, micro granules, sponges, capsules, hydrogels, films, etc. Chitosan membranes [1] are most often obtained by the phase inversion method, i.e., preparation of chitosan salt (e.g., acetate), and then its neutralisation in an aqueous or water/alcoholic alkaline solution or the polycation (chitosan ionic salt)—polyanion system (e.g., sodium tri, hexaphosphate, and sodium alginate). The CS membranes can also be obtained by dissolving chitosan in acetic acid solution at room temperature, and then diluting the obtained solution with water in appropriate proportions. In addition to the many advantages associated with the properties of chitosan, the polysaccharide has some limitations in use. Chitosan in an acidic environment swells strongly under the influence of water and loses its mechanical stability. Increasing the stability of hydrophilic membranes is achieved, e.g., by cross-linking, creating polymer blends or grafting polymers, and modification of their surface [15]. Moreover, the production of asymmetric membranes with a thin active layer deposited on a porous substrate [16] or the creation of hybrid organic–inorganic composites increases the possibilities of their use. The membranes containing particles of inorganic substances in their structure, e.g., zeolites, silica, or metal oxides, are characterised by better transport properties [17]. Moreover, an appropriate amount of a nanocomposite properly distributed in the membrane polymer has a better effect on the improvement of transport–separation properties [8]. Additional benefits resulting from the addition of nanoparticles to the structure of polymer membranes include the modification of the hydrophilicity and electrokinetic potential of the membrane surface, as well as the antibacterial and photocatalytic properties [4]. Thanks to their properties, chitosan membranes are widely used [18] in medicine as dressings, in pharmacology as drug carriers [19], and in biomedical engineering as coatings on implants [16]. CS membranes can also be used for separation of plasma proteins as scaffolds for cell culture and as biocatalyst carriers. In addition, CS membranes are used in membrane techniques, also for metal sorption and water purification [14]. Chitosan, as a low-toxicity hydrophilic biopolymer with additional composite materials, offers a new class of materials for bioimaging applications, for example, as fluorescent beads or a membrane [20,21,22,23]. Polymer composites consist of at least two phases (continuous and dispersed) with distinct interfaces [24]. In nanocomposites and nanocomposite membranes, the dispersed component (nanofiller) has at least one dimension in the nanometric scale (10^−9^ m). In the presented study, fluorescent nanoparticles of fluorinated methacrylate were used as a nanocomposite of chitosan membranes. The morphological properties of the hybrid hydrogel depend on the composite particles, and, consequently, an important parameter is the effective dispersion of particles in hydrogel matrices as beads or membranes. Recently, the trend has been shifted toward utilising polymer NPs in the form of micelles. Cancellation of the charges changes the hydrophilicity of the obtained membrane and the resulting polyelectrolyte complexes with nanoparticles improve its mechanical properties. The combination of hydrophobic and electrostatic interactions results in formation of a unique system from which the dye will not leak out. These interactions make the structure sensitive to variable reaction conditions and, thus, permit a variety of its modifications. Analysis of the obtained new membranes was improved by comparing their properties with those of chitosan membranes modified only with a fluorescent dye and glycerine. The results of this study can help in development of new methods for obtaining hydrogel enriched in acryl nanoparticles, a promising hybrid material that can be used for bioimaging diagnostic purposes [25] or 3D printing [26].

## 2. Materials and Methods

### 2.1. Materials

**Figure 1:**
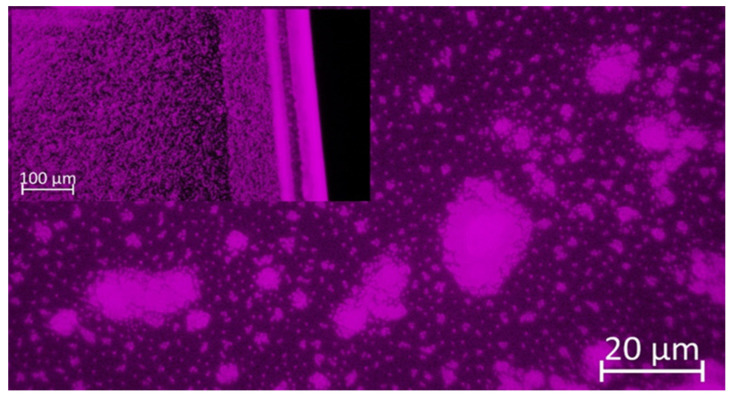
Microscopic image of fluorescent NPsRhB obtained in fluorescence mode ×100. Inserted image magnification ×20 taken at the edge of the measuring chamber.

Chitin from shrimp shells, acetic acid (≥99%), glycerol (≥99.5%), Rhodamine-B-isothiocyanate (Rh B), and sodium hydroxide were purchased from Merc (Poland). Fluorescent poly(heptafluoro-n-butyl methacrylate) particles with Rhodamine B samples (NPs Rh B) were prepared and stored in the authors’ laboratory (in room temperature sample storage). All chemicals and reagents were used as received without any purification. The synthesis, physicochemical characteristics, and long-term stability of these fluorescent nanoparticles were described in the author’s earlier publication and in the articles cited there [27]. Before the incorporation of NPsRhB (hydrodynamic diameter (dH) = 340 nm) into a CS matrix, a microscopic evaluation of fluorescent behaviour was made. Figure 1 presents an image of NPsRhB obtained in fluorescent mode using an inverted microscope (for comparison of the signals from the particles and background, we presented the inserted image magnification taken at the edge of the measuring chamber). Dry particles blend in the measuring chamber but, in higher magnifications, it can be easy to distinguish single particles, as well as agglomerates. It should be mentioned that our previous studies [27] proved the long-term stability of fluorescent behaviour of the used NPsRhB.

The fluorescence behaviour was optically evaluated using an inverted microscope ZEISS Axi-oVert.A1 (Zeiss, Shanghai, China) equipped with a colour camera Axiocam 208 (Zeiss, China) and Colibri 5 system LED light for imaging in fluorescence mode (green LED λ = 555 nm). Imaging was performed using two objectives: LD A-Plan 20×/0,35 (air) and A-Plan ×100/1.25; phase 2 (oil). The sample of NPsRhB was inserted into a 1 μ-Slide VI flat Treat Microscopy Chamber (GmbH, Gräfelfing, Germany). For the presentation, the resolution of the images was automatically adjusted by the best fit with the ZEN3.1 software (Zeiss, Jena, Germany). After confirmation of the fluorescent behaviour of NPsRhB, the particles were mixed with CS solution.

### 2.2. Preparation of Chitosan Membranes

Chitin was placed in a beaker with distilled water in the amount sufficient (0.8 g of chitin in 20 cm^3^ of water) to produce its swelling. A portion of 50 mL of 50% NaOH solution was prepared. One hour later, the swollen chitin was drained and placed in a two-necked flask, and a 50% NaOH solution was added to the flask. The reaction mixture thus obtained was heated for 3 h at 120 °C. After this time, the obtained mixture was filtered on a funnel, washed with distilled water until the filtrate was neutral, and dried at a temperature of about 60 °C. The obtained CS was controlled by solubility test to check its deacetylation degree (by adding a small amount of polymer to 10 cm^3^ of 1 M hydrochloric acid solution and 10 cm^3^ of 1 M acetic acid solution).

Chitosan solution in acetic acid (I). A portion of 4.08 g of chitosan was placed in a 250 cm^3^ single-neck round-bottom flask and 100 cm^3^ of 2.96% acetic acid solution and 2 cm^3^ of distilled water were added. The resulting mixture was stirred for 3 h at room temperature. Thus, a 4% *w*/*v* (weight to volume percentage) solution of chitosan in 3% acetic acid was obtained.

Chitosan solution in acetic acid, diluted 1:4 with water (II). A portion of 14 mL of 4% *w*/*v* chitosan solution I was diluted with 56 mL of distilled water and stirred for half an hour on a magnetic stirrer at room temperature.

Chitosan solutions in acetic acid with the addition of glycerol (III). A portion of 0.11 g of glycerol was added to 20.38 g of chitosan solution II (4% *w*/*v* in 3% acetic acid) and stirred at room temperature for 2 h.

Chitosan solution in acetic acid with the acrylic nanoparticles with Rhodamine B (IV). A portion of 9 mL of chitosan solution II was mixed with 1 mL of the stock solution of NPsRhB (Figure 1) in water. For the experiments, NPs from the stock solution with a concentration of 0.038 g/mL without additional purification were taken.

Chitosan in acetic acid with Rhodamine B nanoparticle (V). A portion of 9 mL of chitosan solution II was mixed with 1 mL of the stock solution of NPsRhB (Figure 1) in water. The mixture was stirred for 3 h at room temperature.

#### Membranes Preparation

Portions of 10 mL of each of the chitosan solutions (II–V) were split into Petri dishes of a diameter of 5 cm. They were left at room temperature for 12–72 h for the water to evaporate and a membrane formation, respectively: CS, CS/Glyc, CS/Rh B, and CS/NPs Rh B. The membrane mass was determined by subtracting the mass of the empty dishes from the mass of the dishes with the membrane.

### 2.3. Morphological and Fluorescent Characterisation of Chitosan Membranes

The membrane thickness, mass, transparency, and surface roughness were determined. Membrane thickness was measured with an electronic digital micrometre (Stainless Hardened Top Craft, Warszawa, Poland) (+/−0.01 mm) at five different positions. The morphologies of the membranes’ surfaces were observed using a stereoscopic microscope (Delta Optical IPOS-810, Nowe Osiny, Poland) with a camera (Delta Optical DLT-Cam 1080 HDMI WIFI 5MP, Nowe Osiny, Poland) and microscopy with epifluorescence module (Delta Optical L-1000, Nowe Osiny, Poland) with BP460–490 (B) and BP510–550 (G) excitation filters. The masses of membranes were determined by Sartorius (+/−0.001 g).

### 2.4. ATR FRIR Spectra Analysis

The attenuated total reflection–Fourier transform infrared (ATR-FTIR) spectrum analysis (JASCO FT/IR-4600 type A with ATR-PRO-ONE and TGS (ABL&E-JASCO Poland, Kraków, Poland)) was applied to characterise the structures of chitosan membranes. The spectra were collected over the range of 300–4000 cm^−1^ with resolution 4 cm^−^^1^ and at a scanning speed of 2 mm/s.

### 2.5. UV-Vis Spectra Analysis

The UV-Vis absorption spectra were recorded using a JASCO V-630 spectrometer, (ABL&E-JASCO Polska Sp. z o.o. Kraków, Poland). The spectra were measured in the range of 200–800 nm at a scan speed of 2 nm/s, with an interval of 1 nm.

### 2.6. Contact Angle (CA) Measurement

The contact angle (+/−0.1°) was measured with a Dataphisics OCA 15EC goniometer (DataPhysics Instruments GmbH, Filderstadt, Germany). Double-sided adhesive tape was used to fix the membrane samples on a measuring slide.

### 2.7. Water Vapour Permeability

Modified upright cup method [28]. Circular fragments of a diameter of 7 mm were cut out from the chitosan membranes and fixed in the holes of the caps to 1.5 mL vials. Each vial with 1 mL of distilled water was left for 7 days and the weight change of the vials were measured. Input conditions of the experiments: air average temperature, 28 °C; air average RH = 37%; and solution temperature, 30 °C. The water vapour transmission rate (WVTR) is defined as:

WVTR = (Wt − W0)/(tA) (gm^−2^d^−1^)

where W0 is the weight of the initial system, Wt is the weight of the system at time t, t is the measuring time, and A is the area of the opening of the vials. Masses of the membranes were determined by Mettler Toledo ME204 (+/−0.0001 g).

### 2.8. Rheological Tests

A ViscoQC 300 viscometer equipped with DIN adapter (Anton Paar GmbH, Graz, Austria) was used to determine the rheological properties of the chitosan solution. The tests were conducted at room temperature using a measuring cup C-CC18 and measuring bob C-CC18. First, speed scan was applied, where the spindle speed was ranged from 7 to 250 rpm, with an increasing trend, which corresponds to a shear rate of 10–322 1/s. Then, after evaluating optimal speed (25 rpm) the measurements were conducted for 10 min.

### 2.9. Formation of Modified Chitosan Beads

A portion of 0.5 g chitosan powder was dissolved in 2% (*v*/*v*) acetic acid/vinegar solution, making the total volume 50 mL. Then, the mixture was slowly added dropwise to 25 mL of 1 M NaOH to form CS beads. The method is similar to [29].

## 3. Results and Discussion

Acetic acid was used to obtain chitosan membrane solutions. The polymer dissolved easily and relatively quickly. If the saccharide copolymer has a deacetylation degree (DD) value above 65%, then the chitosan acetate solution is ready for further use without additional procedures, e.g., filtration. The DD of the obtained chitosan was nearly 90% (value determined by IR spectroscopy [30]). The degree of deacetylation and, thus, the physicochemical properties of the obtained chitosan depend, among other things, on the concentration of the sodium hydroxide solution and its ratio, the time and temperature of the process, the origin of the natural material, and even the atmosphere in which the reaction is carried out [31]. The table below (Table 1) summarises the weights of the chitosan membranes after the complete evaporation of water. Depending on the additives used, the CS membranes had different weights. The membranes with the addition of polymer nanoparticles (from 0.22 g to 0.10 g) are characterised by the greatest mass, while the unmodified chitosan membrane was the lightest (m = 0.07 g). The thickness of the obtained films increased in the order CS < CS/Glyc < CS/Rh B < CS/NPs Rh B and ranged from 20 to 50 μm.

### 3.1. ATR-IR Analysis

**Figure 2:**
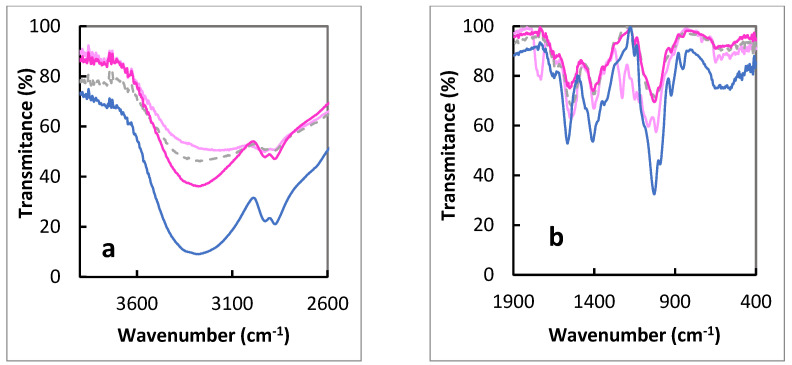
The ATR IR spectra of polymeric film: CS (dashed line), CS/ Gly (blue line), CS/Rh B (dark pink line), and CS/NPs (light pink line) in the region (**a**) 3700−2600 cm^−1^ and (**b**) 1900−400 cm^−1^.

The ATR-IR spectra of chitosan membrane without modification, chitosan membrane with glycerol, chitosan membrane with Rhodamine B, and with the addition of polymer nanoparticles with Rhodamine B are presented below in Figure 2. The spectra on the right of this figure show a characteristic band at approximately 1590 cm^−^^1^, which is attributed to the -NH bending vibration of the primary amine (Table 2). The band at 1150 cm^−^^1^ can be attributed to the asymmetric stretching of the C–O–C bridge. On the other hand, the very intense signals at 1063 and 1024 cm^−^^1^ correspond to the C–O stretching vibrations. The last characteristic band is at 892 cm^−^^1^ and comes from the glucose ring (Table 2). The FTIR spectral bands at 3200–3400 cm^−1^ correspond to ν(O–H) and– -NH_2_ is also involved in intermolecular and intramolecular hydrogen bonds [32]. The differences in the spectra shown in Figure 2 correspond to the presence of different components in CS membranes and changes in the intermolecular and intramolecular hydrogen bonds (Figure 2a).

### 3.2. UV-Vis Analysis—Release of Rhodamine B from Chitosan Membrane Impregnated with the Dye Itself and Encapsulated in Nanoparticles

**Figure 3:**
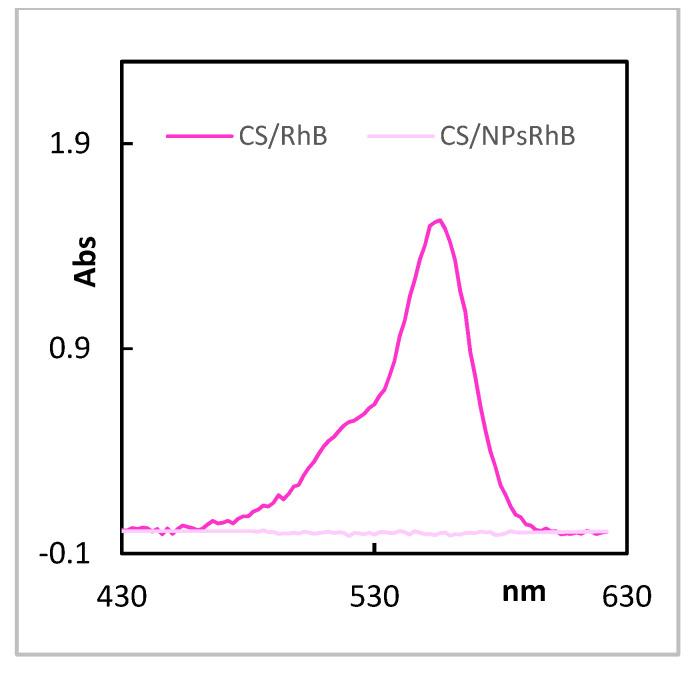
The UV−Vis spectrum of the solution of 0.9% NaCl released Rhodamine B from the investigated membranes.

The information about the chitosan membrane and its affinity to organic compounds was provided by the UV-Vis study. The chosen dye (Rhodamine B) was added directly to the membrane chitosan solution. The formed chitosan films were soaked in 0.9% NaCl so that the dye was gradually released into the solution. The UV-Vis spectrum of Rhodamine B in NaCl solution gives a characteristic band at the wavelength of λmax = 550 nm (Figure 3).

The intensities of this characteristic band recorded for three membrane fragments were similar (A1 = 0.157, A2 = 0.190, A3 = 0.182); therefore, it can be concluded that Rhodamine B was evenly released from the membrane. The Rhodamine B concentration in the tested membrane fragments was determined using the standard curve. The average value of absorption at the wavelength λ = 550 nm was C = 0.176. The Rhodamine B concentrations in the tested membrane fragments were C = 2.013 × 10^−6^ M. The formed chitosan films incorporated with Nps RhB were soaked in 0.9% NaCl in the same way but the dye was not released into the solution. The UV-Vis spectrum of CS/NPs RhB membrane in NaCl solution gives no band at 550 nm (Figure 2) and no dye leakage was observed.

### 3.3. Rough Surfaces of Chitosan Membrane

**Figure 4:**
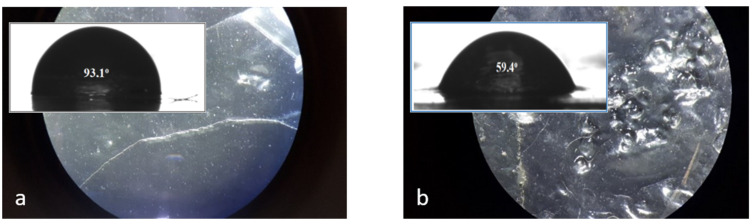

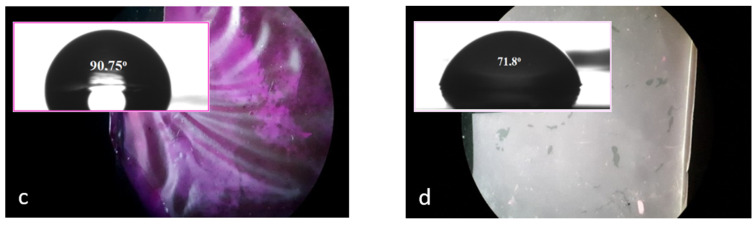
Stereoscopic microscope images and the values of the contact angle (deg) of the modified chitosan hydrogel films (**a**) CS; (**b**) CS/Gly; (**c**) CS/Rh B; and (**d**) CS/NPs Rh B.

The chitosan membranes with various composition modifications were characterized by a stereoscopic microscope and their topographies are shown in Figure 4. The chitosan membrane and the dye-modified membrane (CS and CS/Rh B) had a relatively low roughness of the surface, but the nano-loaded chitosan membranes (CS/NPs Rh B) obtained in this study had a relatively smooth surface. The values of contact angles presented in Figure 4 show that water partially penetrates the grooves of the membrane surface (Figure 4).

**Figure 5:**
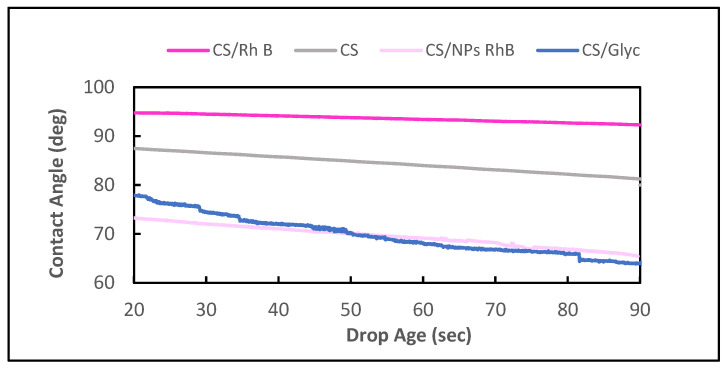
Effect of the ageing time on the wettability of different chitosan films.

The hydrophilic or hydrophobic properties of the chitosan membranes studied were characterised by determination of the contact angle values. The contact angle of unmodified chitosan membrane was 93.1°. The surface modification evidently affected the surface properties of chitosan membranes. An addition of Rhodamine B resulted in a small change in the contact angle, to 90.7° (Figure 4c), whereas the surface of the hydrogel with addition of nanoparticles proved the most hydrophilic of all samples obtained and its contact angle was 71.8°, 12 degrees greater than the contact angle of glycol-modified chitosan membrane, i.e., 59.4°. The CA analysis carried out on the layers is suitable to observe changes in the wetting properties of the surface after the functionalisation process with different atoms [33] or after the addition of compounds with other groups (–OH of glycerol). The time changes in the measured contact angles for all chitosan membranes studied are presented in Figure 5. Evolution of the contact angle with time provides information on both smooth and rough surfaces [34]. Analysis of these data permits evaluation of differences in absorbability of the materials studied.

The initial contact angle is a measure of the membrane surface wetting with water. A high value of contact angle for CS and CS/Rh B indicates poor wetting of their surfaces. Additionally, the greater the change in the contact angle over time, the steeper the slope of the plot (CS/Gly). The slope between 10 s and 20 s of the plot obtained for the CS, CS/Rh B, and CS/NPs Rh B membranes is the same and does not increase much over time. Analysis of the results [35] of the contact angles measured of four membranes has shown that the strength of hydrogel interactions with water depends on the additives, especially types of added nanoparticles.

### 3.4. Water Vapour Permeability Analyses

**Figure 6:**
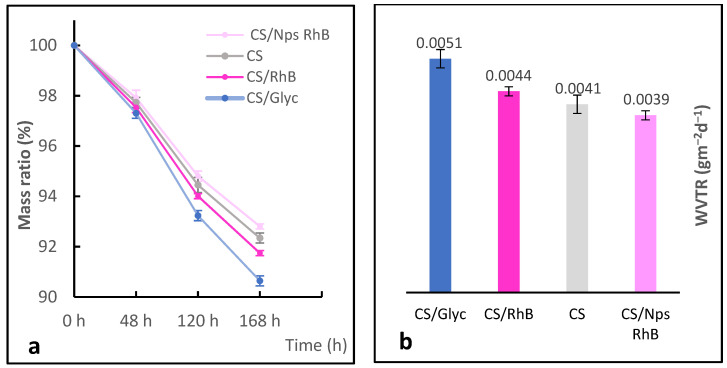
Diffusion of water vapour through the film. Changes in the sample masses as a function of time (**a**) and the water vapour transmission rate (WVTR) (**b**).

The water vapour permeability (WVP) of modified biopolymer films was characterised by a modified upright cup method [28]. The WVP values of chitosan and modified chitosan films are shown in Figure 6. The nanoparticle-loaded chitosan films (CS/NPs Rh B) displayed the best water vapor barrier property of 0.0039 g/m^2^d. CS/Gly had the highest WVP value of 0.0051 g/m^2^d due to the presence of hydrophilic -OH groups in the structure.

**Figure 7:**
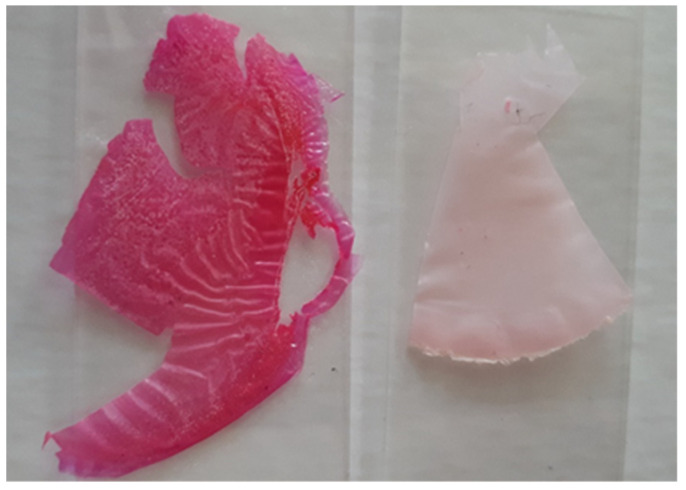
Modified chitosan hydrogel after a few weeks at room temperature: (**left**) chitosan membrane CS/Rh B (**left**) and CS/ NPs (**right**).

The differences in the membrane properties depend mainly on their chemical compositions [4] and, in the samples studied, they are slight. The most pronounced differences in the stability of the foils modified with the dye and the dye trapped in the synthetic polymer appear after their drying (Figure 7).

### 3.5. Formation of Modified Chitosan Beads

**Figure 8:**
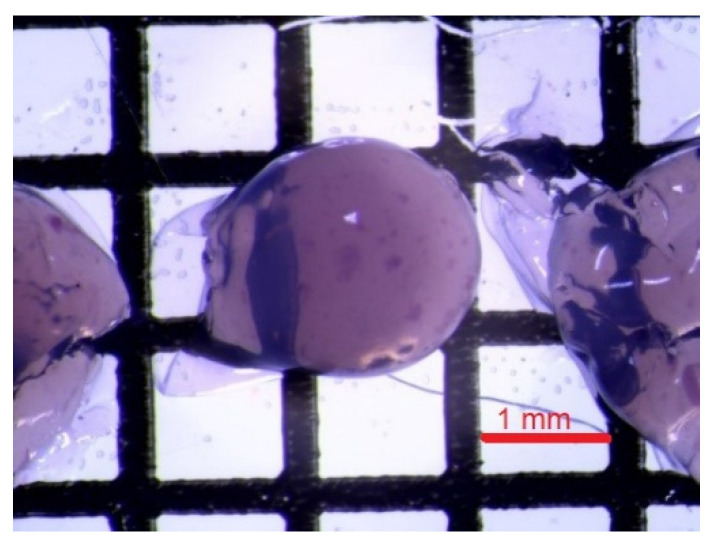
HFBMA NPs in a chitosan hydrogel bead.

The formation of chitosan beads involves the neutralisation of each droplet of chitosan acetic acid solution. Neutralisation is carried out in an alkaline solution. The neutralisation process generates a hydrogel bead that contains water and chitosan copolymer with -NH_2_ group. Often surfactants, such as dodecyltrimethylammonium bromide (DTAB), sodium dodecylsulphate (SDS), or polyoxyethylene lauryl ether (Brij30), are added to the chitosan solution. An increase in the surfactant concentration increases the adsorption potential of the chitosan beads [29,36]. The surfactant molecules may bind with CS in the beads because of the interactions through the chains between the CS backbone and the hydrophobic part of the surfactant and through the polar head of surfactants. In this investigation, we used the water colloidal solution of fluorescent poly(heptafluoro-n-butyl methacrylate) particles (Figure 8). The nanoparticles with long-term stability were synthesised in our group and are ideal for the impregnation of the chitosan. The resulting nanoparticles have a spherical structure, which is the result of the tendency of the molecules to form micellar systems in the reaction mixture. In this type of structures, the hydrophilic heads of the surfactants are directed outwards, while the hydrophobic fragments are part of the internal structure of acrylic nanoparticles. The size of an individual bead was approximately 0.5–1.2 mm.

**Figure 9:**
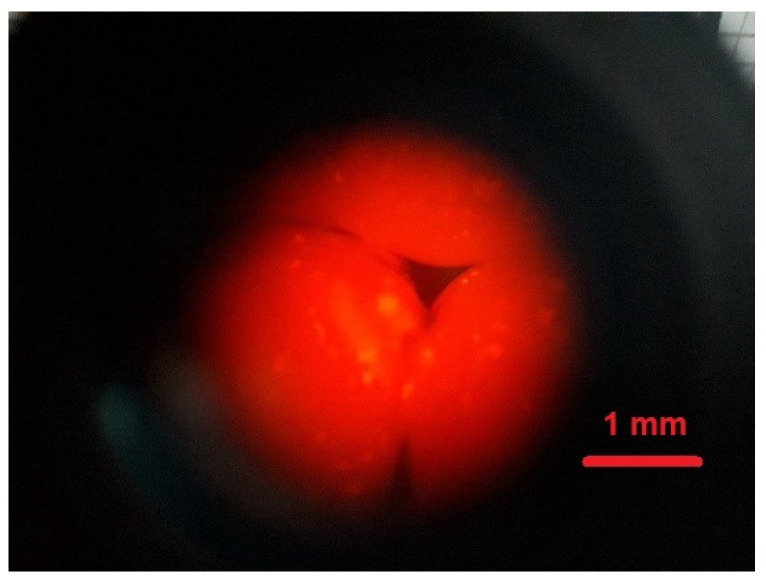
Epifluorescence showing a nonhomogeneous distribution of Rhodamine B dye in different chitosan microcapsules.

The modified chitosan beads showed fluorescence (Figure 9), which confirmed the successful impregnation of NPs into chitosan hydrogel beads.

Core-shell Nps/CS Mps presented in Table 3 contain a fluorescent core with a transparent fluorinated polymer shell, with a refractive index (1.367) [37] close to the water and Rhodamine B (λex 553 nm; λem 627 nm in methanol) as a dye. The main features [38] of fluorescent MPs/NPs used for bioimaging are summarised in Table 3.

**Figure 10:**
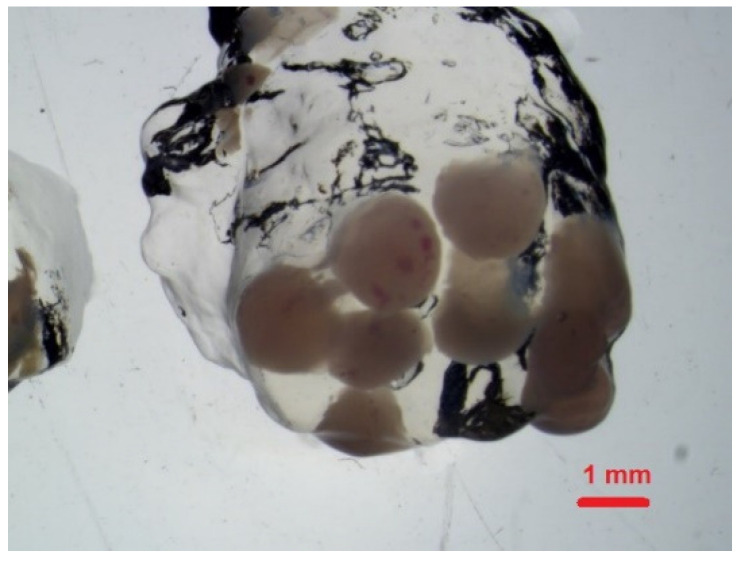
Hybrid MPs in gelatine hydrogel.

These strong interactions cause the attachment of colloidal NPs with chitosan in the beads, thus preventing the release of NPs from the beads during adsorption, as well as during storage in aqueous solutions or in the other medium, for example, gelatine (Figure 10).

**Figure 11:**
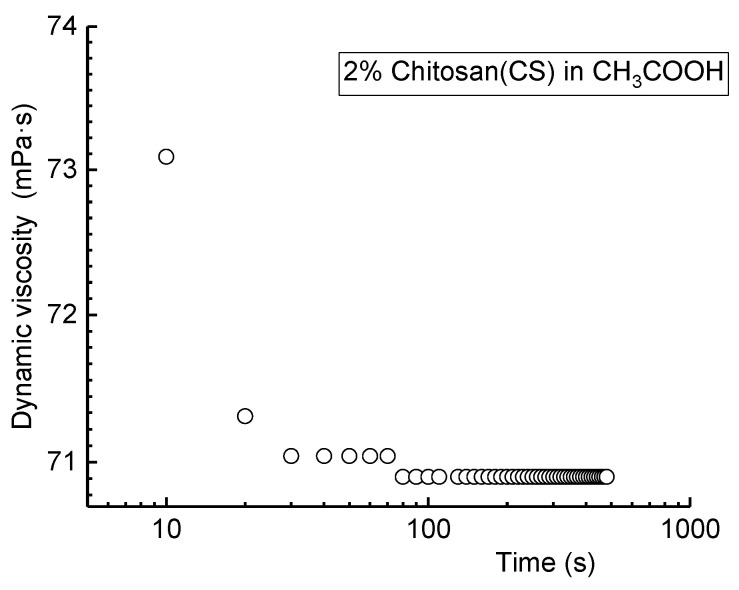
Dynamic viscosity versus time in logarithmic scale for CS solution in 2% acetic acid at 25 rpm spindle.

Taking into account the increasing tendency to use natural polymers for 3D printing [39], it is reasonable to predict that, in the nearest future, the use of chitosan-based inks, in particular, those enriched in fluorescent dyes, will grow. The micelle nanoparticles can effectively form a polyelectrolyte complex based on electrostatic and hydrogen bonds with cationic polysaccharides, leading to better printability of the hydrogel [40]. Rheological behaviour is one of the key factors in the possible applications of biopolymer systems for 3D printing. Figure 11 shows the results of rheological measurements of chitosan in 2% acetic acid as our main CS solution. The mean dynamic viscosity (room temperature) obtained at 25 rpm was 70.9 ± 0.3 (mPa·s). In this study, we focused only on the CS solution without NPs viscosity measurements. (Note that the NPsRhB used in this study tend to agglomerate.) The addition of NPs can change the surface tension, phase behaviour, and rheology of the solution [41]. Further detailed rheological tests should also take into account dedicated printing systems.

## 4. Conclusions

**Figure 12:**
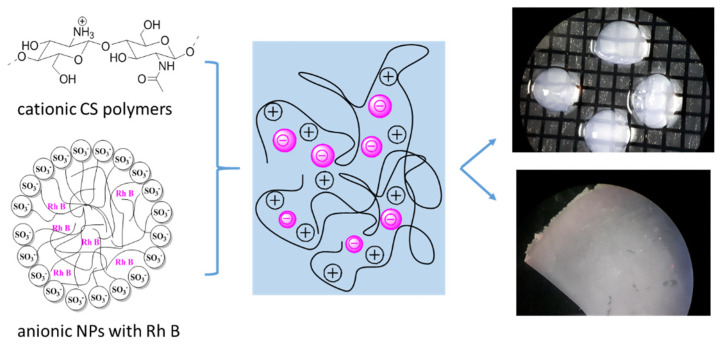
Schematic representation of CS membrane and beads with NPs Rh B-based nanocomposite.

The paper presents a possible strategy for preparation of fluorescent nanoparticle-loaded chitosan hydrogels. The study presents a simple strategy for changing the form of the fluorescent particle-loaded hydrogel depending on its application. In our investigation, the membrane may be in the shape of a flat foil or hydrogel beads (Figure 12).

As follows from the results of physicochemical studies of the materials, the addition of 10% *v*/*v* NPs to a chitosan solution permits a membrane of CA close to 70 degrees to be obtained that remains flexible a long time after preparation. The chitosan beads of the same content of NPs show fluorescent properties and are stable in polar solvents and other gel media, such as gelatine. No leakage of NPsRhB particles from CA membrane film or beads was observed, which is desirable for further applications. In view of CS matrix materials’ possible applications, for example, in bioprinting techniques, further detailed rheological studies should be performed

## Figures and Tables

**Table 1:** Masses and thicknesses ± standard deviation of chitosan films (CS) of different components (Glyc, Rh B, and NPs RH B).

	CS	CS/Glyc	CS/Rh B	CS/NPs Rh B
mas (mg, ±1 mg)	70	80	80	80
thickness (μm, ±10 μm)	20 ± 0.6	30 ± 0.5	40 ± 0.5	50 ± 0.6

**Table 2:** ATR-IR absorption bands and assignments (cm^−1^) of hydrogel films.

(cm^−1^)	CS/Glyc	CS/RhB	CS	CS/Nps RhB
ν(N–H)/ν(O–H), hydrogen bonds	3280–3351	3280–3358	3271–3361	3181–3333
ν(CH_2_)/ν(CH_3_)	2928	2932	2936	2935
ν(C–H)	2874	2875	2878	2877
ν(C=O) (amide I)	1648	1645	1640	1643
δ (N-H) (amide II)	1567	1544	1543	1543
δ (CH_2_)	1408	1400	1403	1400
δ_sym_ (CH_3_)	1377	1378	1375	1377
ν(C–N) (amide III)	1339	1339	1339	1337
ν_asym_ (C–O–C)	1153	1153	1153	1153
ν(C–O)/(C3–OH)			1060	1066
ν(C–O)/(C6–OH)			1020	1018
ν(glucose ring)			900	908

**Table 3:** The main features of fluorescent MPs/NPs proposed for bioimaging.

	Size	Brightness	Photo stability	Detection	Form	Ref.
NPs	(nm)	high	good	color	aqueous suspension	[27]
MPs	(μm)	good	fair	color	NPs-loaded hydrogel	

## Data Availability

The data presented in this study are available on request from the corresponding author.
